# End-to-End One-Shot Path-Planning Algorithm for an Autonomous Vehicle Based on a Convolutional Neural Network Considering Traversability Cost

**DOI:** 10.3390/s22249682

**Published:** 2022-12-10

**Authors:** Tongfei Bian, Yang Xing, Argyrios Zolotas

**Affiliations:** Centre for Autonomous and Cyber-Physical Systems, Cranfield University, Bedford MK43 0AL, UK

**Keywords:** path planning, deep learning, fully convolutional neural network, grid map

## Abstract

Path planning plays an important role in navigation and motion planning for robotics and automated driving applications. Most existing methods use iterative frameworks to calculate and plan the optimal path from the starting point to the endpoint. Iterative planning algorithms can be slow on large maps or long paths. This work introduces an end-to-end path-planning algorithm based on a fully convolutional neural network (FCNN) for grid maps with the concept of the traversability cost, and this trains a general path-planning model for 10 × 10 to 80 × 80 square and rectangular maps. The algorithm outputs the lowest-cost path while considering the cost and the shortest path without considering the cost. The FCNN model analyzes the grid map information and outputs two probability maps, which show the probability of each point in the lowest-cost path and the shortest path. Based on the probability maps, the actual optimal path is reconstructed by using the highest probability method. The proposed method has superior speed advantages over traditional algorithms. On test maps of different sizes and shapes, for the lowest-cost path and the shortest path, the average optimal rates were 72.7% and 78.2%, the average success rates were 95.1% and 92.5%, and the average length rates were 1.04 and 1.03, respectively.

## 1. Introduction

Path planning is defined as the problem of finding a valid state time series from the initial state to the final state under the given constraints [1]. Most path-planning algorithms are good at solving graphic maps with clear and continuous road construction. In such environments, compared with the traversable area, the obstacles of the road are usually buildings or facilities with a large area. Therefore, the traversable area can be abstracted as a continuous road line to improve its planning efficiency. However, when the area of obstacles in the environment is equal to or smaller than the traversable area, such as in a working environment in which a sweeping robot evades the dining table or in which an automated delivery vehicle in a factory evades workers, abstracting the environment into a graphical map will greatly improve the complexity of the map and reduce the planning efficiency. Grid maps can discretize obstacles and traversable areas, so they are suitable and feasible for simulating such working environments and application scenarios at a lower cost and for planning optimal paths in a maze-like environment. In addition, different areas usually require different amounts of time, fuel, or other resources, for example, because of the speed limits in different areas, the fuel consumption of different terrains and road conditions, or tolls for different road sections, which must be considered when planning the optimal path. If closer roads consume more resources, this will be an uneconomic and will provide an unsustainable planning result. Different traversable areas have different costs, which can also be well integrated into grid maps.

Most existing algorithms cannot adapt to grid maps well. Most of them are based on the iterative idea, which leads to extremely low planning efficiency when the grid map is large; they are, thus, unable to achieve real-time planning and have little practical value. For agents in complex work environments, avoiding obstacles and reaching the destination quickly can improve their work efficiency and robustness. In 2020, T. Kulvicius et al. proposed the one-shot method [2], which used fully convolutional neural networks to analyze grid maps, output the probability map of the optimal path, and reconstruct the actual optimal path based on the probability map. Convolutional neural networks are good at processing grid map information, so their planning speed shows obvious advantages. However, the map sizes processed with the one-shot method are small, and do not fully reflect the superiority of its planning speed. In addition, the one-shot method needs different models to plan on maps of different sizes. In addition, one-shot does not consider the cost of different traversable areas.

This work introduces the cost calculation of traversable areas, expands the range of maps that can be planned (including larger map sizes and rectangular map shapes), and trains a general model that can work on all grid map sizes and shapes in the training set and predict the lowest-cost path and the shortest path. The proposed method inherits the speed advantage of the one-shot method and can deal with larger and rectangular maps, handle more flexible application scenarios, solve more complex situations, and obtain better performance, thus providing another feasible solution for path planning on grid maps. The specific contributions of this work are as follows:

1. The concept of the cost of traversable points is introduced. The cost can be understood as the different costs in terms of time, fuel, or other resources for passage through different areas. According to the map information, the proposed method synchronously outputs the lowest-cost path while considering costs and the shortest path without considering costs. Although the path-planning problem is more complex in this case, the application scenario and the meaning of the algorithm are enriched.
2. The maximum size of the map in the database is enlarged to 80 × 80, and rectangular maps with different aspect ratios are generated, thus enriching the map database, reducing the limitations of the method, and giving play to the speed advantage of the FCNN in planning on large maps or long paths.
3. By using the feature that the input matrix of FCNN model can be unfixed, maps of all sizes and shapes in the database are trained at the same time to obtain a general model for all sizes and shapes. The volume of the model is reduced, and the generalization ability and flexibility of the proposed method are improved.
4. The optimal path reconstruction strategy, the highest probability method, is optimized. In this work, when reconstructing a path based on a probability map, the potential optimal location for the next step is filtered by avoiding the obstacles, the passed points, and redundant triangular movements. At the same time, to further improve the success rate of path reconstruction, the rollback mechanism is introduced when the planning fails. The optimization of the path reconstruction strategy significantly improves the success rate of the proposed method.

The structure of this paper is as follows: Section 2 introduces the existing path-planning algorithms, including classical path-planning algorithms such as Dijkstra and A*, sampling-based methods, bio-inspired neural networks, network approaches, and convolutional network path-planning algorithms. Section 3 describes the proposed method in detail, including the experimental design, FCNN design, and path reconstruction method. Section 4 shows the evaluation measures, the effects of different model architectures and parameters, the adjustment of path reconstruction methods on performance, the generalization ability of the proposed method for maps of different sizes and shapes, and a comparison between existing methods and the proposed method. Section 5 discusses the advantages, limitations, and future work. Section 6 summarizes the work of this paper and the performance of the proposed methods.

## 2. Literature Review

### 2.1. Traditional Algorithms

The most common and classic path-planning algorithms are Dijkstra [3] and A* [4]. They are all direct search algorithms for static maps. Dijkstra is a breadth-first divergent search algorithm. It calculates the optimal path from the starting point to all other points until the endpoint is found. A* is a depth-first heuristic algorithm. In an iteration, for each search point, it calculates the distance from the starting point and the expected distance from the endpoint. Some optimization variants [5,6,7,8] improve the efficiency of specific environments or map types. Ref. [9] used A* on hexagonal grid maps. Ref. [10] used Dijkstra and multi-objective optimization algorithms that included the time, cost, and risk to solve the joint decision-making problem of the traffic mode and path in complex networks. Dijkstra and A* can be used in grid maps to plan the optimal path. Dijkstra only considers the traveled distance when planning the optimal path. Therefore, Dijkstra can incorporate the traversable point cost into the distance calculation to plan the lowest-cost path. On the other hand, A* not only considers the traveled distance, but also uses the Manhattan distance to evaluate the estimated distance to the destination to improve the planning speed. Therefore, A* cannot plan the optimal path while considering the traversability cost. So, on grid maps, Dijkstra can plan the lowest-cost path and the shortest path, but A* can only plan the shortest path. In addition, the space and time complexity of traditional algorithms increases dramatically with the growth of the map size/path length [2], resulting in their low planning efficiency and inability to conduct real-time planning on medium and large maps.

### 2.2. Sampling-Based Method

Another common path-planning method is the rapid-exploring random tree (RRT) algorithm [11] and its variants [12,13,14], including RRT* [15], batch notification trees (BIT*-1) [16], and BIT* [17], which have good performance in sparse continuous mapping. In sparse continuous or high-dimensional environments, sampling-based methods have higher computational efficiency in searching paths [11]. However, in a complex maze-like environment, the speed is slowed down due to the “long tail” of their computing time distributions [18]. The idea is to quickly expand a tree structure to explore most areas in an environment and find a feasible path. RRT randomly samples a space and conducts collision tests on the sampling points to avoid the calculation of the accurate modeling of the environment and to improve planning efficiency. However, on complex maze-like grid maps with dense obstacles, their computational efficiency is extremely low and invalid, and the planning results cannot be guaranteed to be optimal [18].

### 2.3. Bio-Inspired Neural Networks

Refs. [19,20,21,22,23,24] used bio-inspired neural networks for path planning. These networks were used to simulate an environment, where the neurons referred to specific points, imitating the locations of cells in the hippocampus [25]. A bio-inspired neural network finds the optimal path by activating the source neuron and transmitting activities and signals to adjacent cells until the target neuron is reached. The path is reconstructed by tracking the active gradient of the network from the source to the target. Bio-inspired neural networks are easier to understand and have adaptability to changes in the environment. However, they are more suitable for planning graphic maps. When they are directly migrated to grid maps, it is necessary to convert the neural network into a grid map, which not only consumes planning time, but also increases the computational complexity because grid maps have fine information granularity. These methods are similar to the traditional algorithms and can ensure that the optimal path is found. However, they require multiple iterations to converge.

### 2.4. Network Approaches

Recently, some methods based on neural network and data-driven approaches have also been proposed, such as shallow networks [26], deep multilayer perceptions [27], long short-term memory networks [28], reinforcement learning [29,30,31,32], and the mixed method [33]. In these methods, an agent predicts the next moving step according to the current state, position, environment, and position of the endpoint until the endpoint is reached. So, they are effective for complex motion planning, such as in robot joint movement, rotation, and expansion [34]. However, for planning paths on grid maps, conceptually, these still use the idea of the iterative algorithms, so they inherit the low efficiency of iterative algorithms on big maps and the disadvantages of machine learning algorithms, that is, the planning result is not guaranteed to be the optimal path.

### 2.5. Convolutional Neural Network Path Planning

When observing a map-planning path, humans always immediately extract the global information from the map, quickly plan a possible and approximate path, and then modify the planning result from the details, instead of repeatedly calculating and comparing the direction of each step from the starting point. This planning ability is defined as perceptual pop-out. When the environment becomes more complex, the time needed for humans to serially search and adjust the path in the planning gradually increases [35]. Ref. [36] found in an experiment that in a complex environment, people scanned the environment repeatedly, and did so more frequently than in a simple environment.

The one-shot method [2] imitated this process. It used an FCNN model to analyze the map information and output a probability map, where the value of each point was the probability of being on the optimal path. Then, according to the probability map, the path reconstruction strategy was used to connect the starting point and the endpoint, thus obtaining the actual optimal path. For grid maps, convolutional neural network path planning (CNNPP) shows strong speed advantages. It subverts the iterative idea of the traditional algorithms and uses an FCNN model to extract the global map information, and its path reconstruction strategy does not accumulate information or calculations for reconstructing the actual optimal path. For maps of different complexities, it has a similar calculation cost. However, the one-shot method can only process maps that are less than 30 × 30, and analyzing different map sizes requires different models. In addition, it cannot analyze the traversability costs.

## 3. Proposed Method

The goal of this work is to predict the optimal paths, avoid all obstacles, and link the starting point and the endpoint for a new environment. Each traversable point in the environment has a corresponding cost value, so the optimal paths include the lowest-cost path while considering the cost and the shortest path without considering the cost. The map of the new environment is not fixed in size and shape, and the location of obstacles, the starting point, the endpoint, and the costs of traversable areas are unseen in the training set. The FCNN model takes the four grid maps corresponding to the obstacle map, cost map, starting-point map, and endpoint map as inputs, and it outputs the probability maps of the two optimal paths. From the input map containing environmental feature information to the output probability maps, it is an end-to-end process that uses raw map information. In a two-dimensional environment, the shapes of the input map and the output map are the same after the convolution calculation when the stride for lateral and vertical movement is set to 1 and the square convolution kernel size *S* and padding *P* meet the following conditions:

(1) S=2×P+1

Then, based on the probability maps obtained from the FCNN model, the highest probability method is used to reconstruct the two actual optimal paths.

### 3.1. Experimental Design

**Figure 1:**
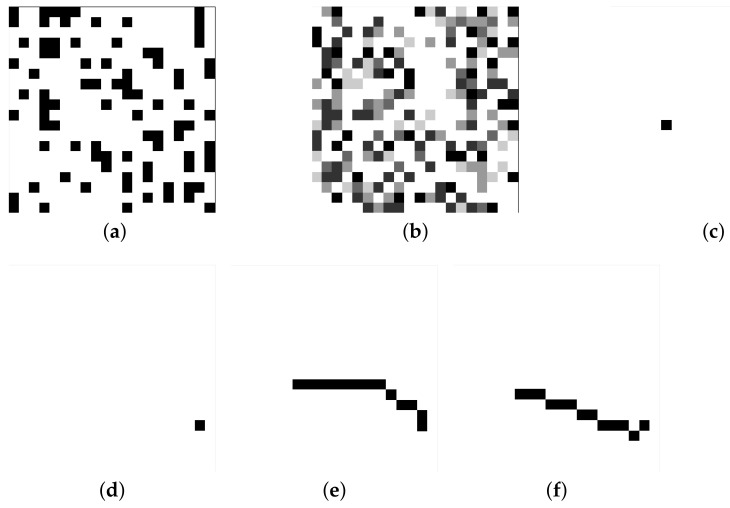
Six dimensions of a map data (**a**): Obstacle map; (**b**): Traversable cost map; (**c**): Starting point map; (**d**): Ending point map; (**e**): Lowest cost path map; (**f**): Shortest path map.

In this work, the grid map’s shapes included rectangles and squares. Its length and width were selected from [10, 20, 40, 60, 80] so that 25 sizes of maps were generated. Obstacles, costs, the starting point, and the endpoint in the maps were randomly generated. Based on the map, the ground truth of the lowest-cost path and the shortest path were obtained by using Dijkstra and A*, respectively. Thus, as shown in Figure 1, each datum had six channels with the shape m × n × 6, where m and n refer to the length and width of the map. Figure 1a–d show the first four input channels, and Figure 1e,f show the last two output channels.

#### 3.1.1. Input Map Generation

**Figure 2:**
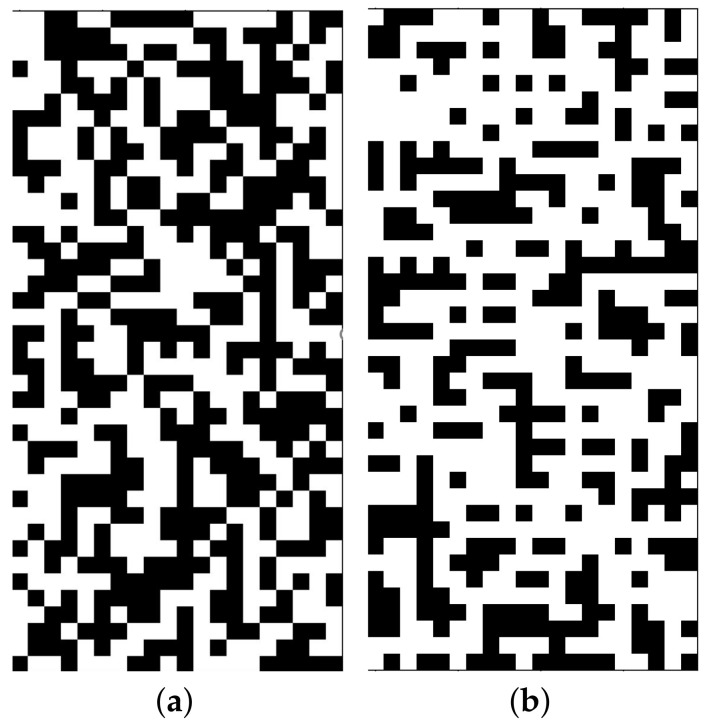
Effect of map adjustment. (**a**) Example of original map; (**b**) Example of adjusted map.

The obstacle maps were binary maps. So, the obstacle points were set to 1 and the traversable points were set to 0. For the obstacle maps, the density of obstacles $P_{O}$ was randomly selected in [0.4,0.6]. In the randomly generated maps, diagonal obstacle structures appeared frequently, as shown in Figure 2a. A diagonal obstacle structure referred to a 2 × 2 area with only two obstacle points, which were placed diagonally. To make the generated grid maps more reasonable and similar to real environments, diagonal obstacles were not allowed in the maps. Because using grid maps to simulate real environments means restoring the positions and shapes of obstacles in the environments with high accuracy, diagonal obstacles are rare in real environments. Moreover, this work allowed the agent to move diagonally, so it could pass through diagonal obstacle structures, which was unreasonable and unrealistic.

To delete the diagonal obstacle structures in the maps, the maps were scanned by column and one of the diagonal obstacles that was found was randomly deleted. To ensure that the density of the obstacles did not change, obstacles were added to the map to offset the loss caused by removing diagonal obstacles. This was done by searching for the positions in the map where obstacles could be placed and would not form a diagonal obstacle structure, randomly selecting one of the available positions to set the obstacles, updating the available positions, and randomly adding an obstacle to the map again until the obstacle density of the current map was equal to that of the original map. If the number of available positions of obstacles was less than the number of removed obstacles, this map would be discarded. An example of an adjusted map is shown in Figure 2b.

The basic cost of each traversable point was 1. The value of the extra cost was randomly selected in the range [0.2,1]. To reduce the computational complexity, only the extra cost values of the traversable points were retained in the cost maps. For the whole map, the generation density of traversable points with extra costs was $P_{c}=0.8-P_{O}$, and the generation position could not coincide with an obstacle position. The density setting was able to ensure that the density of traversable points in the map was dense enough to improve the complexity and diversity of the map. At the same time, it was able to reduce the probability of generating unsolvable maps, thus improving the efficiency of map database generation.

The positions of the starting point and endpoint were randomly selected among the traversable points, and they could not coincide with each other. The starting-point map and endpoint map were also binary maps. The positions of the starting point and endpoint were set to 1 on the corresponding map, and other positions were set to 0. Therefore, only one point in the starting point map and endpoint map had a value of 1.

#### 3.1.2. FCNN Path Planning

The ground truth of the lowest-cost path and the shortest path was obtained by using the Dijkstra and A* algorithms, respectively. A* was faster than Dijkstra when planning the shortest path without considering the cost because the introduction of the estimated distance helped A* filter out many alternative paths that led away from the endpoint. The lowest-cost path map and the shortest path map were binary maps. Each point on the optimal path was set to 1, and the other points were set to 0.

In the process of planning the ground truth, if there was no path linking the starting point and the endpoint due to obstacles, the map was discarded. When the ground truth of a map was obtained but the number of steps in any optimal path was less than $0.2\times (Width_{Map}+Height_{Map})$, the map was discarded. Because the optimal path was too short and a large amount of environmental information was wasted, the model could not learn enough knowledge of path planning from such data. The maps in the database should ensure the complexity of the planning samples as much as possible to improve the learning speed and optimization efficiency of the model.

The Euclidean distance was used in the distance calculation, i.e., for each horizontal or vertical movement, distance=10, and for each diagonal movement, distance=14. When considering the cost, $cost=distance\times cost_{target}$, where $cost_{target}$ is the cost of the target point for each movement.

**Figure 3:**
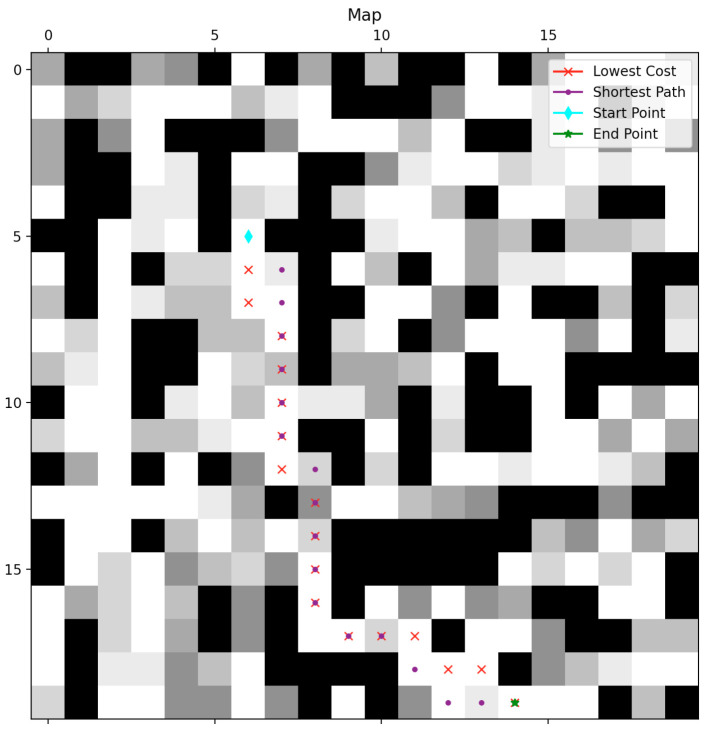
An example of path-planning results for map information and the ground truth.

An example of the map information and path-planning results when using Dijkstra for the lowest-cost path and A* for the shortest map is shown in Figure 3. The black points are obstacles, the white points are traversable points with a basic cost, and the gray points are traversable points with an extra cost. The darker the shade of a gray point, the greater the extra cost of that point. The aqua diamond and the green star are the starting point and the endpoint, respectively. The red-cross path and the purple-dot path are the lowest-cost path and the shortest path, respectively.

#### 3.1.3. Data Requirement

This work increased the maximum map size to 80 × 80. A larger map means more information, and it needs a more complex path-planning process. To avoid creating a huge map database and having a low training efficiency, it was necessary to control the total amount of map data and increase the proportion of larger map data in the database so that the proposed method could perform well on larger maps. At the same time, an FCNN model trained on larger maps could also perform well on smaller maps [2]. Therefore, increasing the proportion of large maps in the training data could enable the model not only to gain more experience in planning the optimal paths on large maps, but also to make use of the knowledge to make up for the shortage of small map data in the database and improve the average performance of the proposed method with all sizes and shapes. At the same time, it could also reduce the possibility of underfitting on large maps and overfitting on small maps.

The length and width of the map were selected from [10,20,40,60,80]. The smallest map size of 10 × 10 had 8000 maps in the database. Each time the length or width of the map shape increased, the number of maps corresponding to that shape increased by 4000. According to this rule, the largest map size of 80 × 80 had 40,000 maps, and the total number of maps in the map database was 600,000.

### 3.2. FCNN Design

#### 3.2.1. Architectures

The input of the model was a four-dimensional map, which included the obstacle map, cost map, starting point map, and endpoint map. The output was a two-dimensional map, which included the probability maps of the lowest-cost path and the shortest path. In this work, a few plain FCNN models and residual networks [37] were trained. The residual network only added the residual connections between convolution layers. So, in general, the residual network had the same characteristics as those of the FCNN model and also met the requirements of this work.

The kernel size of each convolution layer of these models was 3 × 3, and the padding was set to SAME to ensure that the input and output map shapes were the same, except for the dimension-reducing and dimension-increasing convolution layers in the bottleneck structure of the residual network, whose convolution kernel size was 1 × 1 and whose padding was set to VALID. The activation function of the last convolution layer for outputting the probability maps was Sigmaid. The activation function of the other convolution layers is ReLU, which increased the nonlinearity and improved the efficiency.

In the plain FCNN models, a batch normalization layer was added after each convolution layer in order to accelerate model convergence and avoid gradient disappearance and gradient explosion, and a 10% dropout layer was added at the end to avoid overfitting.

In the residual networks, to ensure that the shape of the output map was the same as that of the input map, all of the max-pooling layers were deleted, the kernel size of the first convolution layer was changed from 7 × 7 to 3 × 3, and the padding was set to SAME. For the residual network to output two probability maps, the number of kernels in their last convolution layer was set to 2, the size of the kernels was set to 3 × 3, and the padding was set to SAME. Section 4 will show a performance comparison of the plain FCNN and ResNet models with different layers and kernels.

#### 3.2.2. Training Procedure

For each map shape in the map database, 300 maps were randomly selected as the validation set, 200 maps were selected as the test set, and the rest were used in training sets. Selecting the same number of maps for each shape to compose the validation set and test set instead of selecting maps of the same proportion allowed fair calculation of the average performance of the model on all map shapes during validation and testing.

The models used the mean squared error (MSE) between the output and the ground truth as the loss function. This was because the MSE converged quickly and made the training process stable. All of the output target maps of this work were the ground truth, so there were no outliers, which allowed the shortcomings of the MSE to be avoided. At the same time, the MSE imposes large penalties on large errors and small penalties on small errors, which was also applicable to this work. The MSE was able to improve the success rate of the model and reduce the length ratio as soon as possible, rather than consuming many computing resources to get too close to the ground truth because the optimal path was not unique.

In addition, Adam was used as the optimizer of the models, as it could make the models converge quickly in the training process, but it could also cause the models to oscillate near the optimal solution. Similarly, it was not desired in this work for the model to excessively pursue making the output the same as the ground truth.

Due to the large volume of the models and the map database, 20 maps were trained for each batch. If the average length ratio of planning the lowest-cost path and the shortest path on the validation set did not increase in 5 epochs during the training process, the training process was considered finished with the corresponding parameters and stopped. The length ratio represented the difference between the planning path and the optimal path, which is defined in Section 4

### 3.3. Path Reconstruction

**Figure 4:**
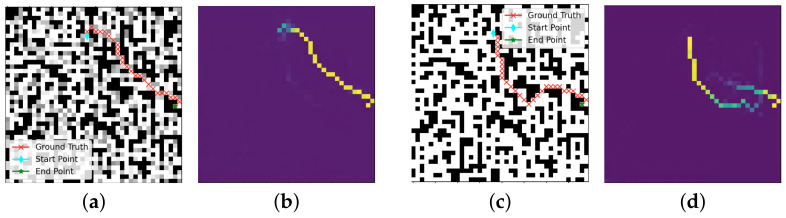
Example of the ground-truth, environment, and probability maps. (**a**) Ground truth and environment of the lowest-cost path of the sample. (**b**) Probability map of the shortest path of the sample. (**c**) Ground truth and environment of the shortest path of the sample. (**d**) Probability map of the shortest path of the sample.

After analyzing the obstacles, costs, starting point, and endpoint, the model output two probability maps, as shown in Figure 4. The highest probability method was used to obtain the actual optimal paths according to the probability maps. The strategy was to compare the probability of the points around the current point, select the point with the highest probability as the next step position, and move the current point to that position for the next comparison and selection. The planning process started from the starting point and the endpoint at the same time until the two paths intersected. The intersection point could be the starting point or the endpoint. When the two paths intersected, the paths from the intersection point to the starting point and endpoint were connected to form the optimal path predicted by the proposed method.

**Figure 5:**
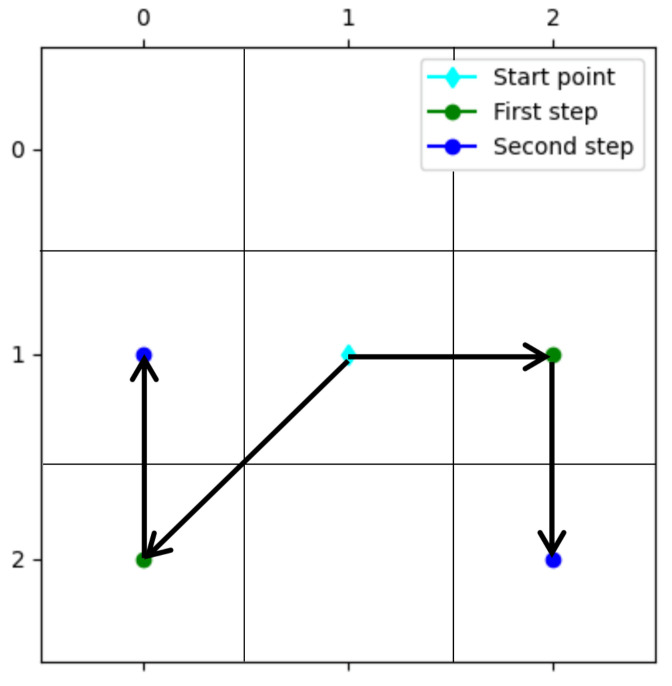
Examples of redundant triangular movements.

To make the strategy of the highest probability method more reasonable, this work filtered the available positions of each next step. Firstly, the locations of obstacles were ignored to avoid paths being predicted by the models crossing obstacles. Secondly, positions that had been traveled were ignored. The points in the traveled path usually had a high probability, so ignoring them prevented the planned path being stuck with the point with the highest probability in the probability map, rather than the actual destination. Finally, positions that caused redundant triangular movements were ignored. A redundant triangular movement refers to two consecutive movements by which the position reached after the movement can be replaced by a single movement. There are two cases of redundant triangular movements—two mutually perpendicular horizontal and vertical movements can be replaced by a single diagonal movement, and a diagonal movement and a vertical or horizontal movement with an included angle of 45 degrees can be replaced by another horizontal or vertical movement, as shown in Figure 5. In the process of path reconstruction, avoiding redundant triangular movements could prevent the large virtual distance and cost caused by inefficient planning results and could improve the planning effect and speed of the model.

In path reconstruction, if the current point was surrounded by a map boundary, obstacles, traveled points, or points that will cause redundant triangular movement, then there were no available points around the current point for the next movement, thus causing the path reconstruction to fail. To improve the performance of path reconstruction, in this case, the path reconstruction strategy would be rolled back, which involved changing the corresponding probability of the current point on the probability map to 0 and backtracking the position of the current point to the position of the previous point in the planned path. Then, when searching for the next moving location, the points that caused the failure of path planning were ignored. The rollback operation was able to improve the success rate of path planning. However, the number of consecutive rollbacks should be limited. Otherwise, the path reconstruction process would compare and consider many points with low probabilities on the probability map, which would not only consume much time and computation costs, but also would not improve the performance.

## 4. Results

### 4.1. Evaluation Measures

In this work, the success rate, optimal rate, and length ratio were used as evaluation measures to measure and realize the performance of the proposed method. To compare the planning efficiency between the proposed method and other methods, the evaluation measures also included the planning speed.

#### 4.1.1. Success Rate

If the model obtained a path that could connect the starting point and the endpoint, the path planning was considered successful. The success rate SR was calculated as the percentage of successful planning results in all test environments:

(2) $$SR=100\%\times \frac{N_{S}}{N_{E}}$$

where $N_{E}$ is the total number of maps analyzed by the model and $N_{S}$ is the number of maps for which the model could successfully plan the optimal path.

#### 4.1.2. Optimal Rate

The planning result was considered optimal when the distance or cost of the path prediction was the same as that of the ground truth. Since there could be different optimal solutions for the same map, the optimal solution could not simply be determined by comparing the predicted path with the ground truth. The optimal rate OR was computed as the percentage of optimal planning results out of all tested environments:

(3) $$OR=100\%\times \frac{N_{O}}{N_{E}}$$

where the $N_{E}$ is the total number of maps analyzed by the model and the $N_{O}$ is the number of maps for which the model could plan paths with the same distance or cost as those of the ground truth.

#### 4.1.3. Length Ratio

When the planning was successful but had a large difference in distance or cost from the optimal path, the planning result was not helpful or useful. The length ratio shows the difference between the planning result and the ground truth. The length ratio LR was computed as the ratio of the distance or cost of the planning result to the distance or cost of the ground truth:

(4) $$LR=\frac{Length_{Predict}}{Length_{GroundTruth}}$$

where $Length_{GroundTruth}$ is the distance or cost of the optimal path according to the ground truth, and $Length_{Predict}$ is the distance or cost of the planning result.

#### 4.1.4. Planning Speed

The planning speed Speed refers to the number of steps that the model can plan in one second. The faster the planning speed is, the higher the real-time performance of the algorithm is and the more application scenarios there are. The planning speed is computed as the ratio between the total steps of the planning result and the planning time:

(5) $$Speed=\frac{Steps}{Time}$$

where Steps is the number of steps in both the lowest-cost path and the shortest path and Time is the sum of the time taken to plan the lowest-cost path and the shortest path.

### 4.2. Parameter Analysis

All of the success rates, optimal rates, length ratios, and planning speeds in the parameter analysis were calculated according to the average performance of the corresponding model in the test set composed of 25 × 200 maps. The test set had 25 map shapes, and each map shape had 200 maps. This work compared the effects of parameters in the plain FCNN model, residual network model, and path reconstruction strategy on the performance of the proposed method in order to select the optimal parameters and model.

#### 4.2.1. Plain FCNN Model

Two hyperparameters of the plain FCNN models were tested: the number of layers and the number of kernels per layer. The number of kernels in each layer remained the same. As shown in Table 1, the optimal model was a 36-layer fully convolutional neural network with 96 3 × 3 kernels per layer.

#### 4.2.2. Residual Network

The residual network can solve the degradation problem caused by network depth. Residual blocks can enable redundant blocks to learn identity mapping when a network has stacked too many blocks, thus ensuring that the performance will not decline and the model will not degenerate. Therefore, the actual depth of the network is determined in the training process, that is, a residual network has the ability to make its depth self-adaptive. Residual network models with 18, 34, 50, and 101 layers were selected for training in this work. ResNet18 and ResNet34 used the basic block residual structure, and ResNet50 and ResNet101 used the bottleneck residual structure. As mentioned above, to meet the requirements of this work, the residual network had the maximum pooling layer removed from the original structure, and parameters in the first and last convolution layers were adjusted. The residual network structure used in this work is shown in Table 2. As shown in Table 3, the optimal model was ResNet34.

#### 4.2.3. Path Reconstruction Strategy

In the process of path reconstruction, when selecting the next movement position around the current point, it was obvious and understandable to ignore the obstacles and the travelled positions. Therefore, Table 4 only shows the contributions to the avoidance of redundant triangular movements when using ResNet34. It can be seen from the comparison of before and after that avoiding redundant triangular movements greatly improved the success rate, optimal rate, and length ratio of the model.

This work introduced a rollback mechanism for the path reconstruction strategy in order to deal with situations in which the current point was surrounded by non-selectable points, which would lead to the failure of path planning. However, if the agent was allowed to roll back without a limited number of times, it would not only fail to further improve the model performance, but also reduce the planning speed of the model. To ensure that the performance and the planning speed of the model were optimal, this work tested different upper limits of the number of consecutive fallback times, as shown in Table 5.

As shown in Table 5, when the upper limit of the number of rollbacks was 4, the model performance was improved to the upper limit. To ensure the model planning speed, 4 was selected as the optimal limit of the number of consecutive rollbacks. The rollback mechanism could improve the success rate, but because more difficult maps resulted in non-optimal solutions, the average length ratio slightly decreased. The rollback mechanism had little effect on the optimal rate.

### 4.3. Generalization Capability

**Figure 6:**
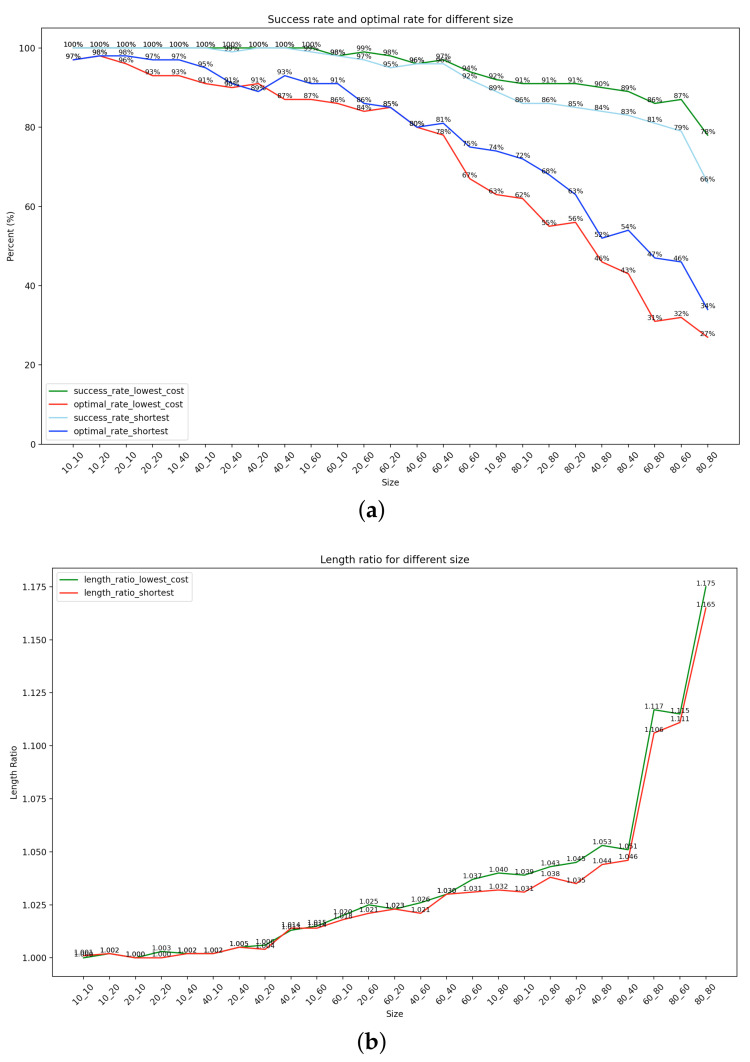
(**a**): Success rate and optimal rate of different sizes; (**b**): Length ratio of different sizes.

As shown in the comparison above, ResNet34 was the optimal model. Its success rates and optimal rates on each map shape are shown in Figure 6a, and its length ratios on each map shape are shown in Figure 6b.

It can be seen from the figures that, on all map shapes, the success rate of the lowest-cost path and the shortest path of the model planning was high, and even on larger maps, the success rate could generally be around 80%. The optimal rate of the two optimal paths predicted by the model was also more than 60% on small and medium-sized maps. The length ratio of the two optimal paths predicted by the model was less than 1.175, which meant that the total cost or distance of almost all successfully planned paths was less than 117.5% of the actual optimal path. This showed that the performance of the model was excellent, effective, and useful.

### 4.4. Comparison with Other Methods

#### 4.4.1. Performance Comparison

The performance of the proposed method was compared to that of other path-planning methods, including RRT [11], BIT*-1 [16], and the one-shot method [2], as shown in Table 6. All of these methods were run on the same test set, which was composed of 200 × 25 maps. The traditional algorithms could always plan the optimal paths, so they were ignored in the performance comparison. For RRT, the upper limit of the search samples was set to 3000, and the probability of sampling the endpoint was set to 10%. The sampling distance was set to 2, which meant that each sampling movement was two horizontal or vertical movements in the same direction or one diagonal movement, which kept the path from the current point to the sampling point unique and allowed a cost calculation. For BIT*-1, the batch size was set to 1500, and the sampling distance was also set to 2. For both RRT and BIT*-1, if the endpoint was not found when using all of the samples, the planning failed. For the one-shot method, the FCNN structure and path reconstruction strategy in [2] and the same training data as those used for the proposed method were used.

Compared with the FCNN methods, RRT and BIT*-1 did not perform well on grid maps with dense obstacles. So, it was difficult for them to find a path that linked the starting point and endpoint due to limitations in the samples and sampling distance. Therefore, the optimal rate of BIT*-1 was lower than that of the FCNN methods. RRT was more concerned with finding the endpoint than the optimal path, so its optimal rate was extremely low. Compared with the proposed method, the one-shot method has a simpler model structure and path reconstruction strategy, so its performance was poorer and its planning speed was faster.

#### 4.4.2. Speed Comparison

**Figure 7:**
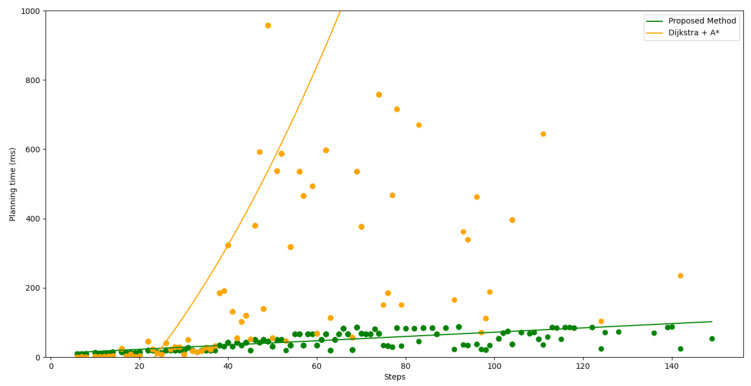
Speed comparison between the traditional algorithms and the proposed method.

Although BIT*-1 and the one-shot method were faster than the proposed method, their planning performance was obviously poorer. So, only the traditional algorithms composed of Dijkstra and A* were used for the speed comparison with the proposed method. This work predicted the lowest-cost path and the shortest path in new environments. Therefore, the planning speed of the proposed method needed to be compared with that of Dijkstra and A* together. As shown in Figure 7, the proposed method and the traditional algorithms were run on 25 × 20 maps. For each map, the corresponding steps and planning time were the sum of planning the lowest-cost path and the shortest path. The planning time started from the traditional algorithms or the proposed method that received the map information to output the actual prediction paths. For each map, the sum of the steps of the two optimal paths and the running time for planning using the traditional algorithms and the proposed method are shown by orange dots and green dots respectively. A straight line was used to fit the relationship between the planning time and the steps of the proposed method. For the traditional algorithms, conic fitting is used. To better show the intersection of two lines, only the points with a planning time of less than one second are shown in the figure, so most of the results that were planned by using the traditional algorithms are cut off.

When the number of steps in an optimal path was larger than 30, compared with the traditional algorithms composed of Dijkstra and A*, the proposed method had a great speed advantage. When the number of steps to be planned was longer, which also meant that the map was larger, the speed advantage was more obvious. The proposed method had two efficient processes in the path-planning process. The first process was the use of ResNet34 to analyze the map information and get the probability maps. A 34-layer residual network requires less computation and time to process a four-channel matrix that is less than or equal to 80 × 80. The second process was based on the probability maps, and the highest probability method was used to reconstruct the actual optimal paths. This process only needed to collect the information in a small area around the current point for calculation and comparison and to plan the next step in that area. The reconstruction process did not accumulate information and computations. Therefore, compared with the traditional algorithms, the proposed method had obvious advantages in the planning speed.

## 5. Discussion

### 5.1. Advantages of the Proposed Method

The proposed method greatly improved the planning speed while ensuring the usefulness ofthe planning results. This work expanded the maximum map size to 80 × 80, introduced rectangular maps to expand the solvable map shapes, and trained a general model that could be used for all map shapes smaller than the maximum map size. In addition, this work introduced the concept of cost in a map, so that when the model predicted the shortest path, it also needed to analyze the cost in the map to predict the lowest-cost path. At the same time, the performance of the proposed method was better than that of other planning methods, except for Dijkstra and A*, but its planning speed was much faster than that of the traditional algorithms.

### 5.2. Constraints of the Proposed Method

While a novel contribution, the proposed method—at this point—addresses the planning of the optimal paths on static maze-like grid maps that include a single starting point and endpoint. For other map types, it will run more slowly and perform more poorly than other path-planning algorithms. In addition, the proposed method was used in application scenarios that could be represented as grid maps, such as the motion planning of autonomous vehicles and the path planning of intelligent robots in specific spaces other than urban traffic. It is noted that in grid maps, the traversable area aligns with the area of obstacles/restraints. In the case of urban navigation, path planning addresses route selection instead of the avoidance of obstacles.

### 5.3. Future Work

Future work will concentrate on the attempt to obtain a more complex FCNN model structure—rather than adding more training data—to improve the results. It was noted that with more data, the model performance had limited improvement. The adjusted highest probability method still may not be the optimal path reconstruction strategy. Because of the huge advantage in the planning speed, more complex strategies can be introduced for path reconstruction to optimize the model’s performance. When analyzing the information around the current point to find the optimal position for the next step, the scope of information collection can be expanded, or more complex convolutional neural networks, recurrent neural networks, or reinforcement learning models can be introduced to analyze the probability maps.

In addition to cost, height is also environmental information that can be easily integrated into grid maps and can be used as a new channel of maps to make planning more meaningful and complex so as to enrich the practical significance of CNNPP algorithms. Since the planning speed of the proposed method for each map was less than 100 ms, its real-time planning capabilities can be used to plan the optimal path in a dynamic environment. Moreover, it is useful to consider multiple starting points and ending points in a map. This relates to application scenarios in which the environmental information remains static, except for the starting points and endpoints. This could further accelerate the planning speed.

This work proposed an approach looking at advanced path planning for vehicle platforms while taking the traversability cost into account. The particular interest in addressing a vehicle going through map features (normally stemming from the processing of known ordnance survey maps) that emphasize traversability. In the current literature, there is work addressing peripheral areas in the development of future mobility, i.e., self-driving and autonomous cars. The authors of [38] discussed a lightweight and more accurate sensing system, VIPS, while those of [39] referred to the AoI (Age of Information) to enable the optimization of sensor task scheduling for the real-time perception of the environment. These form useful examples that can inform the proposed approach in order to enhance how more variable features are dealt with. In addition, the authors of of [40] referred to realistic operation scheduling (i.e., self-driving taxis), which defined the mission for the agent to perform. This also showcases the potential of the proposed concept in integration with enriching application scenarios in a sequence.

## 6. Conclusions

In this paper, a new CNNPP algorithm, which used a residual network to analyze map information to obtain probability maps and used the highest probability method to reconstruct the actual optimal paths based on the probability maps, was proposed. After testing and comparison, ResNet34 was used to analyze the map information to obtain probability maps, and the highest probability method, which ignored redundant triangular movements and set a limitation of 4 for consecutive rollbacks, was used to reconstruct the actual optimal paths. Among all of the map shapes, when the proposed method planed the lowest-cost path, the average success rate was 95.1%, the average optimal rate was 72.7%, and the average length ratio was 1.04. When the proposed method planned the shortest path, the average success rate was 92.5%, the average optimal rate was 78.2%, and the average length ratio was 1.03. The average planning speed of the proposed method was 3009 steps per second. In most maps, the proposed method was able to successfully plan the path linking the starting point and the endpoint, and because the length ratio was close to 1, this showed that the planned path was close to the optimal path, so it was useful and meaningful. This algorithm considered the traversability cost in path planning, and compared with the traditional algorithms, the proposed method had obvious advantages in terms of planning speed.

## Figures and Tables

**Table 1:** Performance of the plain FCNN models.

With 64 Kernels per Layer	With 31 Layers	With 36 Layers	With 41 Layers
Success rate of the lowest-cost path	75.6%	85.1%	48.0%
Optimal rate of the lowest-cost path	31.7%	49.3%	19.0%
Length ratio of the lowest-cost path	1.10	1.07	1.18
Success rate of the shortest path	75.3%	83.6%	44.6%
Optimal rate of the shortest path	40.5%	63.6%	18.8%
Length ratio of the shortest path	1.10	1.07	1.16
**With 96 kernels per layer**			
Success rate of the lowest-cost path	87.6%	89.4%	88.3%
Optimal rate of the lowest-cost path	38.1%	54.7%	42.2%
Length ratio of the lowest-cost path	1.11	1.06	1.09
Success rate of the shortest path	85.2%	88.6%	87.9%
Optimal rate of the shortest path	55.2%	69.3%	65.3%
Length ratio of the shortest path	1.11	1.06	1.09
**With 128 kernels per layer**			
Success rate of the lowest-cost path	82.2%	84.7%	79.4%
Optimal rate of the lowest-cost path	32.9%	34.6%	29.6%
Length ratio of the lowest-cost path	1.14	1.13	1.16
Success rate of the shortest path	79.8%	81.7%	74.7%
Optimal rate of the shortest path	44.7%	46.9%	41.2%
Length ratio of the shortest path	1.14	1.11	1.14

**Table 2:** Structure of the residual networks.

Layer Name	ResNet18	ResNet34	ResNet50	ResNet101
Conv1	3 × 3, 64	3 × 3, 64	3 × 3, 64	3 × 3, 64
Conv2_x	$\left[\begin{array}{cc} 3\times 3, & 64\\ 3\times 3, & 64 \end{array}\right]$× 2	$\left[\begin{array}{cc} 3\times 3, & 64\\ 3\times 3, & 64 \end{array}\right]$× 3	$\left[\begin{array}{cc} 1\times 1, & 64\\ 3\times 3, & 64\\ 1\times 1, & 256 \end{array}\right]$× 3	$\left[\begin{array}{cc} 1\times 1, & 64\\ 3\times 3, & 64\\ 1\times 1, & 256 \end{array}\right]$× 3
Conv3_x	$\left[\begin{array}{cc} 3\times 3, & 128\\ 3\times 3, & 128 \end{array}\right]$× 2	$\left[\begin{array}{cc} 3\times 3, & 128\\ 3\times 3, & 128 \end{array}\right]$× 4	$\left[\begin{array}{cc} 1\times 1, & 128\\ 3\times 3, & 128\\ 1\times 1, & 512 \end{array}\right]$× 4	$\left[\begin{array}{cc} 1\times 1, & 128\\ 3\times 3, & 128\\ 1\times 1, & 512 \end{array}\right]$× 4
Conv4_x	$\left[\begin{array}{cc} 3\times 3, & 256\\ 3\times 3, & 256 \end{array}\right]$× 2	$\left[\begin{array}{cc} 3\times 3, & 256\\ 3\times 3, & 256 \end{array}\right]$× 6	$\left[\begin{array}{cc} 1\times 1, & 256\\ 3\times 3, & 256\\ 1\times 1, & 1024 \end{array}\right]$× 6	$\left[\begin{array}{cc} 1\times 1, & 256\\ 3\times 3, & 256\\ 1\times 1, & 1024 \end{array}\right]$× 23
Conv5_x	$\left[\begin{array}{cc} 3\times 3, & 512\\ 3\times 3, & 512 \end{array}\right]$× 2	$\left[\begin{array}{cc} 3\times 3, & 512\\ 3\times 3, & 512 \end{array}\right]$× 3	$\left[\begin{array}{cc} 1\times 1, & 512\\ 3\times 3, & 512\\ 1\times 1, & 2048 \end{array}\right]$× 3	$\left[\begin{array}{cc} 1\times 1, & 512\\ 3\times 3, & 512\\ 1\times 1, & 2048 \end{array}\right]$× 3
Conv6	3 × 3, 2	3 × 3, 2	3 × 3, 2	3 × 3, 2

**Table 3:** Performance of the residual networks.

Layers	ResNet18	ResNet34	ResNet50	ResNet101
Success rate of the lowest-cost path	81.0%	90.1%	81.7%	88.8%
Optimal rate of the lowest-cost path	45.5%	70.6%	43.5%	59.7%
Length ratio of the lowest-cost path	1.10	1.05	1.09	1.07
Success rate of the shortest path	77.3%	88.6%	75.6%	87.0%
Optimal rate of the shortest path	47.0%	76.6%	46.2%	66.7%
Length ratio of the shortest path	1.09	1.05	1.08	1.06

**Table 4:** Effect of avoiding redundant triangular movements.

Performance	Before	After
Success rate of the lowest-cost path	90.1%	92.7%
Optimal rate of the lowest-cost path	70.6%	73.5%
Length ratio of the lowest-cost path	1.05	1.03
Success rate of the shortest path	88.6%	91.7%
Optimal rate of the shortest path	76.6%	79.1%
Length ratio of the shortest path	1.05	1.03

**Table 5:** Performance of different limits of rollbacks.

Performance	3	4	5
Success rate of the lowest-cost path	94.2%	95.1%	95.3%
Optimal rate of the lowest-cost path	72.9%	72.7%	72.6%
Length ratio of the lowest-cost path	1.04	1.04	1.04
Success rate of the shortest path	92.1%	92.5%	92.7%
Optimal rate of the shortest path	78.5%	78.2%	78.1%
Length ratio of the shortest path	1.03	1.03	1.04
Planning speed (steps/s)	3053	3009	2924

**Table 6:** Performance of different path-planning methods.

Performance	RRT	BIT*-1	One-Shot	Proposed Method
Success rate of the lowest-cost path	76.3%	71.8%	82.3%	95.1%
Optimal rate of the lowest-cost path	3.3%	13.7%	35.4%	72.7%
Length ratio of the lowest-cost path	1.45	1.32	1.24	1.04
Success rate of the shortest path	68.1%	65.2%	76.9%	92.5%
Optimal rate of the shortest path	5.8%	18.1%	44.2%	78.2%
Length ratio of the shortest path	1.42	1.30	1.21	1.03
Planning speed (steps/s)	1074	5439	7866	3009

## Data Availability

Not applicable.

## Abbreviations

The following abbreviations are used in this manuscript:

FCNN	Fully convolutional neural network
CNNPP	Convolutional neural network path planning
RRT	Rapidly exploring random tree
BIT	Batch-informed tree
AOI	Age of Information
